# Characterization and expression patterns of a membrane-bound trehalase from *Spodoptera exigua*

**DOI:** 10.1186/1471-2199-9-51

**Published:** 2008-05-20

**Authors:** Bin Tang, Xiaofei Chen, Yang Liu, Honggang Tian, Jian Liu, Jian Hu, Weihua Xu, Wenqing Zhang

**Affiliations:** 1 State Key Laboratory of Biocontrol and Institute of Entomology, School of Life Sciences, Sun Yat-sen University, Guangzhou, PR China

## Abstract

**Background:**

The chitin biosynthesis pathway starts with trehalose in insects and the main functions of trehalases are hydrolysis of trehalose to glucose. Although insects possess two types, soluble trehalase (Tre-1) and membrane-bound trehalase (Tre-2), very little is known about Tre-2 and the difference in function between Tre-1 and Tre-2.

**Results:**

To gain an insight into trehalase functions in insects, we investigated a putative membrane-bound trehalase from *Spodoptera exigua *(SeTre-2) cloned from the fat body. The deduced amino acid sequence of SeTre-2 contains 645 residues and has a predicted molecular weight of ~74 kDa and p*I *of 6.01. Alignment of SeTre-2 with other insect trehalases showed that it contains two trehalase signature motifs and a putative transmembrane domain, which is an important characteristic of Tre-2. Comparison of the genomic DNA and cDNA sequences demonstrated that *SeTre-2 *comprises 13 exons and 12 introns. Southern blot analysis revealed that *S. exigua *has two trehalase genes and that *SeTre-2 *is a single-copy gene. Northern blot analyses showed that the *SeTre-2 *transcript is expressed not only in the midgut, as previously reported for *Bombyx mori*, but also in the fat body and Malpighian tubules, although expression patterns differed between the midgut and fat body. *SeTre-2 *transcripts were detected in the midgut of feeding stage larvae, but not in pupae, whereas *SeTre-2 *mRNA was detected in the fat body of fifth instar larvae and pupae.

**Conclusion:**

These findings provide new data on the tissue distribution, expression patterns and potential function of membrane-bound trehalase. The results suggest that the *SeTre-2 *gene may have different functions in the midgut and fat body.

## Background

The disaccharide trehalose consists of two α-glycosidically linked glucose units. It is a non-reducing sugar found in many organisms as diverse as bacteria, yeast, fungi, nematodes, plants, insects and some other invertebrates, but is absent in mammals [1-4]. Trehalose may serve as a carbohydrate store and as an agent for protecting proteins and cellular membranes from a variety of environmental stress conditions, including desiccation, dehydration, heat, freezing and oxidation [5,6]. In plants, trehalose not only has an impact on some metabolic processes and affects plant development as a signaling molecule, but also serves as an anti-stress substance to protect plants from drought, high salt and low temperature [2,7]. In insects, unlike in mammals, trehalose is the main blood sugar and is present in the hemolymph of larvae, pupae and adults [1,8-11]. It is the main reserve sugar in the hemolymph of flying insects and is also indispensable for thermotolerance in larvae.

Trehalose is synthesized mainly in the insect fat body and is rapidly released into the hemolymph and other tissues. To utilize blood trehalose, insect tissues contain trehalases (EC 3.2.1.28) that catalyze the hydrolysis of one mole of trehalose to two moles of glucose. Thus, for uptake or utilization of trehalose in the blood, trehalases are essential enzymes in insects and are thought to be located on the cell membrane or within cells [8,12-15]. The first insect trehalase, a soluble trehalase, was reported in 1992 [16]. Although insects are believed to have two types, soluble trehalase (Tre-1) and membrane-bound trehalase (Tre-2) [16-22], the *Tre-2 *gene was not reported until 2005 [21]. In *Bombyx mori*, the Tre-2 gene is expressed in the midgut; immunoblotting and immunohistochemical analyses showed that Tre-1 is present mainly in goblet cell cavities and Tre-2 penetrates the cell membrane and is predominantly evident on visceral muscle surrounding the midgut [21]. Although two trehalase genes have been cloned from *B. mori*,*Apis mellifera *[22] and *Spodoptera exigua*, the different functions of these two trehalases in chitin biosynthesis in insects are not clear. In addition, very little is known about the structure, tissue distribution and expression pattern of Tre-2.

Here, we report our findings regarding the gene (*SeTre-2*) coding for a putative membrane-bound trehalase isolated from the fat body of *S. exigua *(GenBank EU106080). We observed that it is expressed not only in the midgut, but also in the fat body and Malpighian tubules. Furthermore, its expression patterns differed between the midgut and fat body.

## Results

### Cloning of full-length *SeTre-2 *cDNA

**Figure 1:**
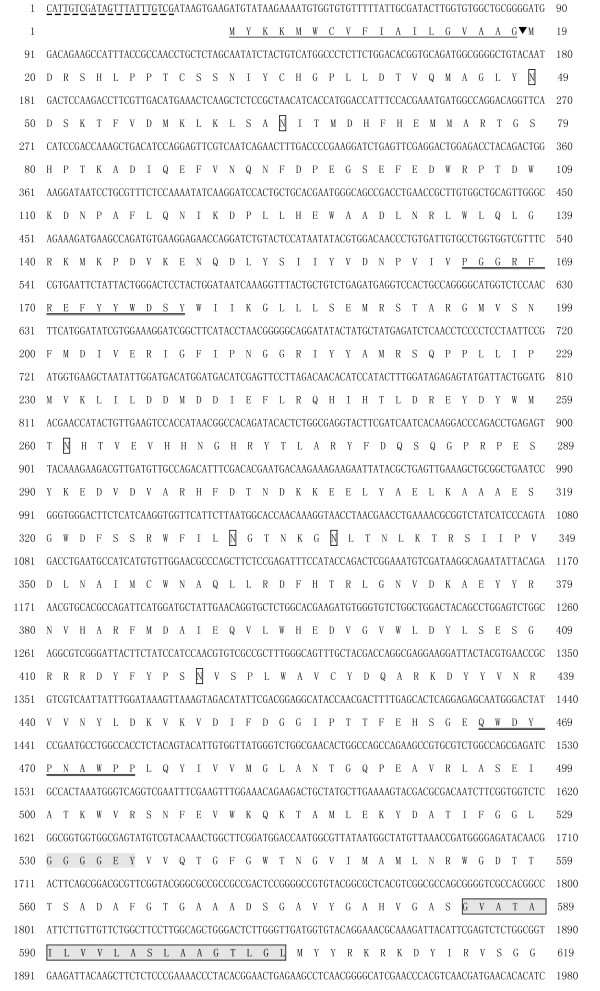
**Nucleotide and amino acid sequences of Tre-2 from the beet armyworm *S. exigua***. Underlined amino acid residues (1–18) and the arrowhead represent the signal peptide and putative cleavage site, respectively. Trehalase signature motifs (amino acid residues 165–178 and 466–475) are double underlined. The highly conserved glycine-rich region is shaded. The putative transmembrane region (residues 585–607) is shaded and boxed. Potential N-glycosylation sites (amino acid residues 49, 64, 261, 331, 337 and 419) are boxed. The nucleotide sequence reported in this paper has been submitted to GenBank under accession number EU106080.

Based on the conserved amino acid and nucleotide sequences of trehalases from *B. mori *(*BmTre-1*, *BmTre-2*), *Tenebrio molitor *(*TmTre-1*) and *Pimpla hypochondriaca *(*PhTre-1*), we designed three degenerate primers, SeTre-F1, SeTre-F2 and SeTre-R, for PCR reactions. A fragment of 690 bp was first obtained from pupal fat body cDNA by a second PCR using SeTre-F2 and SeTre-R. The deduced amino acid sequence exhibited high similarity to insect trehalase sequences. We then performed 5' and 3' rapid amplification of cDNA ends (RACE) using several specific primers based on the sequence of the fragment and universal primers (Clontech). PCR products of 1200 and 600 bp were amplified by 5' and 3' RACE, respectively. Assembly of the overlapping fragments revealed a full-length cDNA of 2195 bp. The trehalase contained an open reading frame of 1938 bp, encoding a protein of 645 amino acids (Figure 1) with a predicted molecular weight of approximately 74 kDa and p*I *of 6.01.

**Figure 2:**
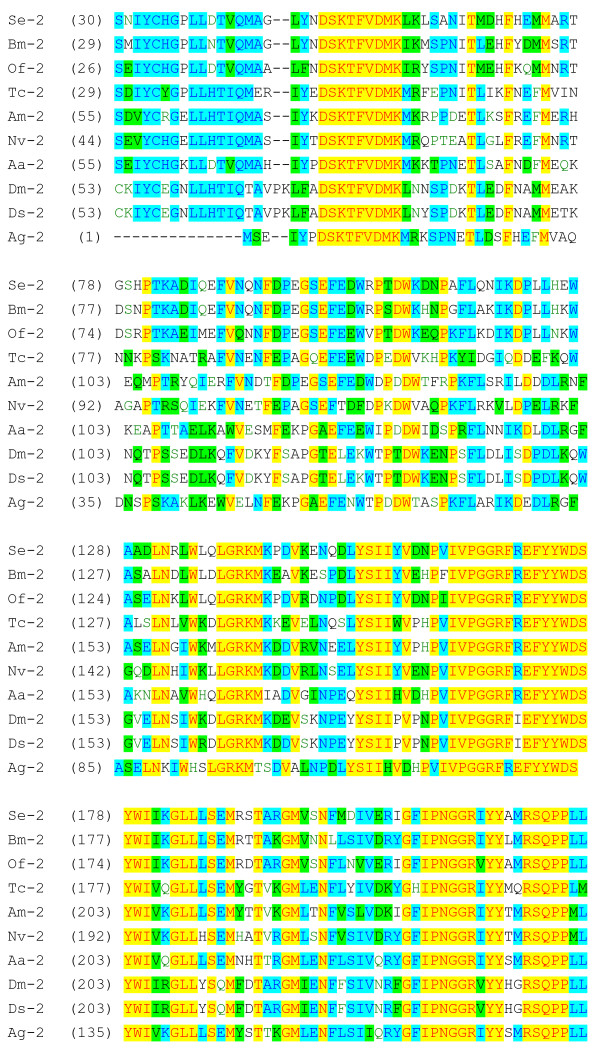
**Alignment of deduced amino acid sequences coded by the *Tre-2 *gene in insects**. Alignment of deduced amino acid sequences coded by *Ag-2 *(GenBank accession no. XP_320471), *Aa-2 *(EAT38444), *Se-2 *(EU106080), *Bm-2 *(ABH06695), *Nv-2 *(XP_001602179), *Of-2 *(EF426723), *Am-2 *(XP_394271), *Tc-2 *(XP_972610), *Ds-2 *(ABH06710) and *Dm-2 *(ABH06695) using Vector NTI 9.0 multiple sequence alignment software. Highly conserved regions are highlighted in yellow and blue.

**Figure 3:**
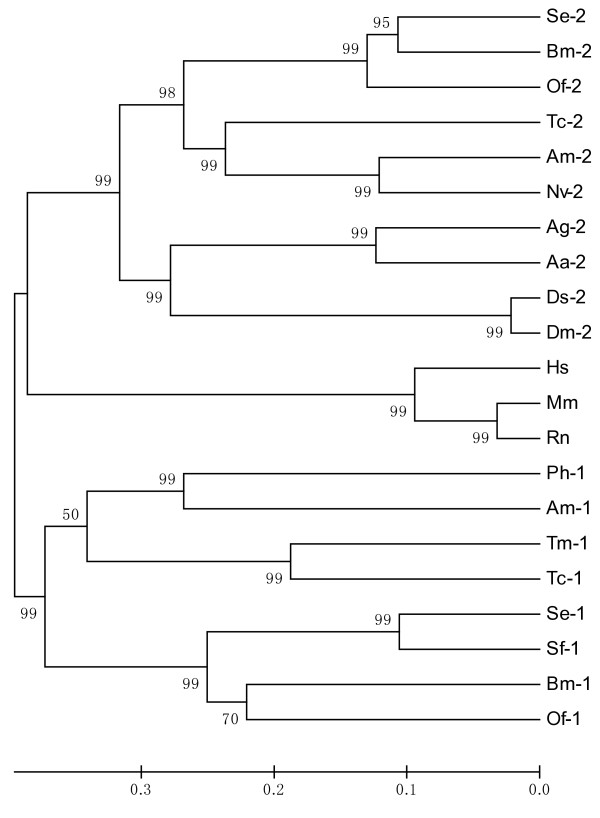
**Phylogenetic analysis of insect trehalases based on amino acid sequences**. Full-length amino acid sequences were aligned using the Mega 3.1 program to generate a phylogenetic tree (1, class 1 gene of soluble trehalase; 2, class 2 gene of membrane-bound trehalase). A bootstrap analysis was carried out, and the robustness of each cluster was verified in 1000 replications. The scale on the x-axis represents estimated branch lengths and numers indicate bootstrap values. Trehalases were from *Aedes aegypti *(Aa), *Anopheles gambiae *(Ag), *Apis mellifera *(Am), *Bombyx mori *(Bm), *Drosophila melanogaster *(Dm), *Drosophila simulans *(Ds), *Homo sapiens *(Hs), *Mus musculus *(Mm), *Nasonia vitripennis *(Nv), *Omphisa fuscidentalis*(Of), *Pimpla hypochondriaca *(Ph), *Rattus norvegicus *(Rn), *Spodoptera exigua *(Se), *Spodoptera frugiperda *(Sf), *Tribolium castaneum *(Tc) and *Tenebrio molitor *(Tm). GenBank accession numbers (DNA) are as follows: *Aa-2*, EAT38444; *Ag-2*, XP_320471; *Am-1*, XM_393963; *Am-2*, XP_394271; *Bm-1*, BAA13042; *Bm-2*, AB162717; *Dm-2*, ABH06695; *Ds-2*, ABH06710; *Hs*, NM_007180; *Mm*, NM_021481; *Nv-2*, XP_001602179; *Of-1*, EF426742; *Of-2*, EF426723; *Ph-1*, Q8MMG9; *Rn*, CH473975; *Se-1*, EU427311; *Se-2*, EU106080; *Sf-1*, ABE27189; *Tc-1*, XP_973919; *Tc-2*, XP_972610; *Tm-1*, P32359.

The deduced amino acid sequence of trehalase from *S. exigua *was aligned with the corresponding sequences of other insect trehalases (Figure 2). SeTre-2 is most similar to lepidopteran trehalase-2 from *B. mori *(BmTre-2; 77% identity) and *Ostrinia furnacalis *(OfTre-2; 76%). It is also similar to SeTre-1 (40%), SfTre-1 (41%), BmTre-1 (44%), OfTre-1 (43%), PhTre-1 (44%), AmTre-2 (54%), AmTre-1 (44%), TcTre-2 (54%), NvTre-2 (50%), AgTre-2 (50%), AaTre-2 (47%), DmTre-2 (45%), DsTre-2 (45%), TmTre-1 (44%), TcTre-1 (46%), RnTre (44%), MmTre (44%) and HsTre (45%). The insect *Tre-2 *gene is highly conserved, particularly in the middle of the putative catalytic domain (Figures 2 and 3).

### *SeTre-2 *cDNA and protein sequence analysis

The deduced amino acid sequence of SeTre-2 contains two trehalase signature motifs, PGGRFREFYYWDSY (residues 165–178) and QWDYPNAWPP (466–475) (Figures 1 and 2) and five other conserved motifs: DSKTFVDMK (residues 50–58), IPNGGRIYY (210–218), RSQPPLL (221–227), GPRPESYKEDV (284–294) and ELKAAAESGWDFSSRWFI (312–329). Residues 1–18 are a signal peptide leader and residues 530–536 correspond to a glycine-rich region (Figure 1). Residues 585–607 were found to comprise a putative transmembrane domain. N-terminal to this domain, residues 573–575 (Ser-Gly-Ala) are identical to amino acids 570–572 (Ser-Gly-Ala) in BmTre-2. However, this is not identified as an omega site by the big-PI predictor used to predict glycosylphosphatidyl inositol modification sites [21,23]. Five potential N-glycosylation sites (amino acids 48, 63, 260, 330 and 336) are present in BmTre-2, but six potential N-glycosylation sites were found in SeTre-2, five sites (amino acids 49, 64, 261, 331 and 337) homologous to those in BmTre-2 and an additional site at amino acid 419.

### Structure of *SeTre-2*

**Figure 4:**
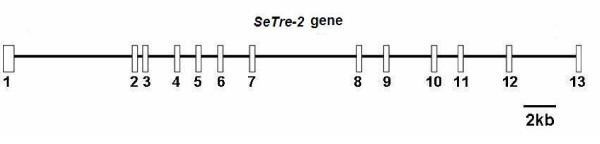
**Trehalase gene in *S. exigua***. The *SeTre-2 *gene comprises 13 exons (boxes with numbers) and 12 introns. The positions of the start and termination codons located 276–278 of the first exon and 129–131 of the last exon in the nuclear acid sequence, respectively. The length of the 13 exons is 364, 141, 116, 160, 167, 155, 194, 171, 177, 216, 111, 143 and 237 bp, separated by 12 introns of various lengths of 5490, 310, 1451, 635, 757, 1282, 4755, 1034, 1751, 1008, 2170 and 2808 bp, respectively.

We amplified the *SeTre-2 *genomic DNA sequence, which is approximately 26 kb long. The exon/intron composition of the gene was determined by comparing the genomic sequence with the *SeTre-2 *cDNA sequence. The *SeTre-2 *gene consists of 13 exons separated by 12 introns of different lengths and exon-intron splice junctions following the GT-AG rule (Figure 4). The first intron is the longest, at 5.5 kb. Exons 1–13 correspond to nucleotides 1–364, 5855–5995, 6306–6421, 7873–8032, 8668–8864, 9592–9746, 11,029–11,222, 15,869–16,038, 17,074–17,250, 19,002–19,217, 20,226–20,336, 22,507–22,649, and 25,518–25,751 in the genomic sequence, respectively. Compared to the seven exons and six introns of the *A. mellifera Tre-2 *gene [22] and the nine exons and eight introns of *AmTre-1 *(data from NCBI), *SeTre-2 *has more exons and introns.

### Southern blot analysis

**Figure 5:**
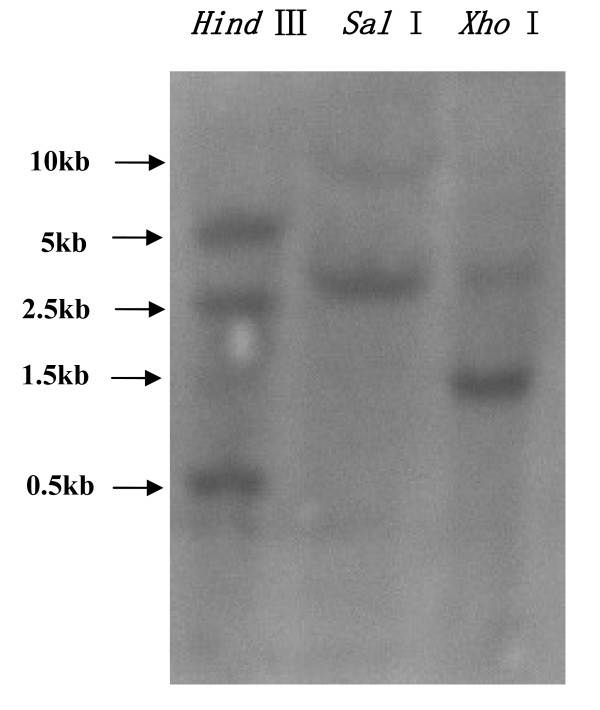
**Southern blot analysis of *S. exigua *genomic DNA**. Samples (15 μg) of *S. exigua *genomic DNA were digested with *Hin*dIII, *Sal*I or *Xho*I. DNA fragments were separated by electrophoresis, transferred onto a nylon membrane, and hybridized with an [α-^32^P]dCTP-labeled *SeTre-2 *cDNA fragment. The strong and faint bands correspond to the *SeTre-2 *and *SeTre-1 *genes, respectively. The numbers on the left are DNA ladder sizes.

Gene copy number can be determined by Southern blot analysis. Genomic DNA was obtained from *S. exigua *pupae and approximately 15 μg of DNA was digested with *Hin*dIII, *Sal*I and *Xho*I, electrophoresed and transferred to a nylon membrane, and then probed with a *SeTre-2 *cDNA fragment (~770 bp) generated using SeTreFP and SeTreRP primers. The probe was designed to be specific for a highly conserved region so that it would hybridize to both *SeTre-1 *and *SeTre-2*. One strong and one faint band were detected when genomic DNA was digested with *Sal*I and *Xho*I (Figure 5) whereas three strong and two faint bands were observed for *Hin*dIII treatment. The latter is attributed to the presence of two *Hin*dIII sites in the *SeTre-2 *genomic sequence that correspond to the probe sequence. A *Hin*dIII site may also be present in the *SeTre-1 *genomic sequence corresponding to the *SeTre-2 *probe.

### *SeTre-2 *tissue distribution

**Figure 6:**
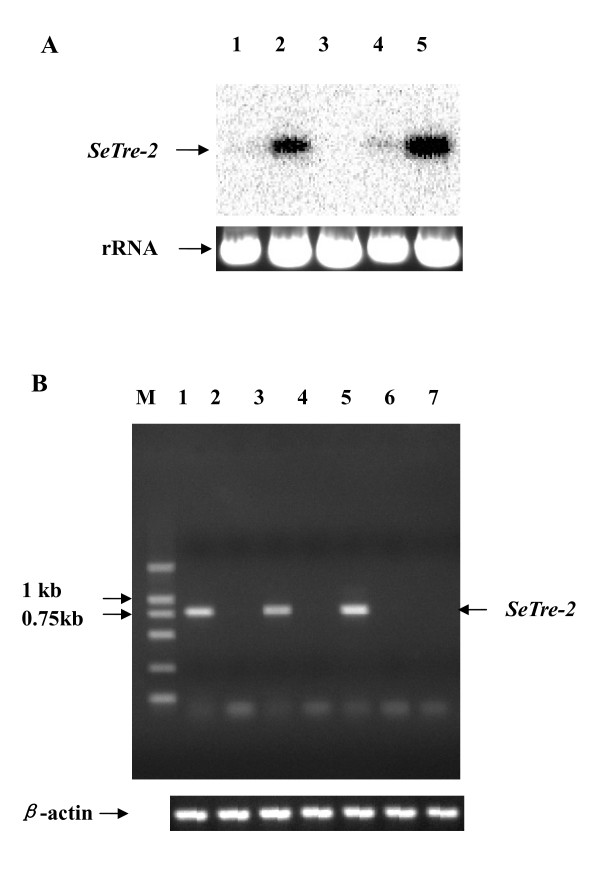
**Northern blot (A) and RT-PCR (B) analyses of the *SeTre-2 *transcript in different tissues of fifth instar larvae of *S. exigua***. **(A) **For Northern blot analysis, total RNA was extracted from various tissues: brain (1), fat body (2), cuticle (3), Malpighian tubules (4), and midgut (5). Probes specific for *SeTre-2 *were radiolabeled using [α-^32^P]dCTP. **(B) **RT-PCR analysis of DL2000 marker (M), midgut (1), brain (2), Malpighian tubules (3), cuticle (4), fat body (5), trachea (6), and spermary (7). β-Actin was used a loading control and visualization was by ethidium bromide staining.

Tissue-specific expression *SeTre-2 *was determined by Northern blotting. *SeTre-2 *cDNA was cloned from *S. exigua *fat body, suggesting that *SeTre-2 *mRNA is expressed in this tissue. This was confirmed by Northern blot analysis (Figure 6A). In addition, *SeTre-2 *transcripts were also detected in the midgut, but not in the brain or cuticle (Figure 6A). *SeTre-2 *mRNA may also be expressed in Malpighian tubules, since a faint band was observed. To determine the expression of *SeTre-2 *transcripts in Malpighian tubules, RT-PCR was performed and a product of the size predicted for the *SeTre-2 *transcript was observed in Malpighian tubules. Sequencing results confirmed the RT-PCR result (Figure 6B), demonstrating that *SeTre-2 *is expressed in the fat body, midgut and Malpighian tubules.

### Developmental *SeTre-2 *expression

**Figure 7:**
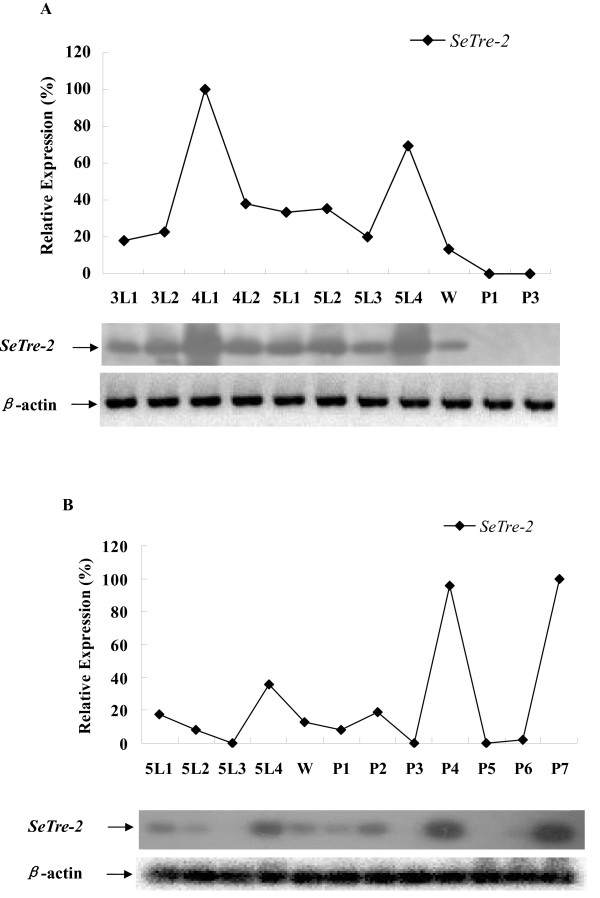
**Developmental expression of *S. exigua Tre-2 *mRNA in the midgut (A) and fat body (B)**. [α-^32^P] dCTP-labeled *SeTre-2 *cDNA was amplified using specific primers SeTreFP and SeTreRP and used as a probe. β-Actin was labeled with [α-^32^P]dCTP as a control.**(A) **RNA was extracted from the midgut third instar (3L), fourth instar (4L), fifth instar (5L) and wandering (pre-pupae) larvae (W) and from pupae (P). **(B) **RNA was extracted from the fat body of fifth instar (5L) and wandering (pre-pupae) larvae (W) and pupae (P).

Semi-quantitative RT-PCR experiments were carried out to determine *SeTre-2 *expression patterns in the midgut and fat body during different developmental stages of *S. exigua. SeTre-2 *transcripts were detected in the midgut of larvae throughout the feeding stage, with higher expression levels in day-1 fourth instar (Figure 7A, lane 3) and day-4 fifth instar larvae (lane 8). However, no *SeTre-2 *expression was observed in the midgut of day-1 and day-3 pupae (lanes 10 and 11). Furthermore, *SeTre-2 *expression was observed in the midgut of pre-pupae (Figure 7A). In fat body, *SeTre-2 *expression patterns were different. *SeTre-2 *mRNA was detected in the fat body of fifth instar larvae and pupae (Figure 7B). Furthermore, higher *SeTre-2 *expression levels were observed in the fat body of day-4 fifth instar larvae (lane 4), as well as day-4 and day-7 pupae (lanes 9 and 12). Lower *SeTre-2 *expression levels were observed in the fat body of day-1 and day-2 fifth instar larvae, pre-pupae, and day-1 and day-2 pupae. However, *SeTre-2 *expression was not observed in the fat body of day-3 fifth instar larvae and day-3, day-5 and day-6 pupae.

## Discussion

Two types of trehalase, soluble (Tre-1 or acid trehalase) and membrane-bound trehalase (Tre-2 or neutral trehalase), have been purified from a variety of organisms, and the corresponding genes have also been cloned. Trehalases facilitate the uptake and utilization of trehalose from food or blood [10-12,24-29]. Insects also have two types of trehalases [16-19,21,22,30,31]. The presence of two trehalase genes in *S. exigua *was verified by Southern blotting (Figure 5). We cloned one trehalase gene and protein sequence analysis suggested that it codes for a soluble trehalase (GenBank accession no. EU427311). These results are consistent with studies in other insect species for which trehalase genes have been cloned [16-19,21,22,30-32].

An insect trehalase gene was first cloned from *Tenebrio molitor *[16,33]. Trehalase genes in *B. mori *[17,18] and *P. hypochondriaca *[30] have also been cloned and studied. All of these insect genes code for soluble trehalases and are expressed mainly in the pupal midgut [16-19,30,31], but also in larval midgut, Malpighian tubules and ovary [17,18]. Although immunoblot analysis revealed that two trehalases exist in insects [12], the second trehalase gene, *Tre-2*, was not reported until 2005 for *B. mori *and 2007 for *A. mellifera *[21,22]. The *BmTre-2 *gene is completely different from the *Tre-1 *gene of *B. mori *[17,18] and *T. molitor *[16,33]. *BmTre-2 *transcripts are expressed in the midgut of *B. mori *larvae [21]. The *Tre-2 *gene structure was first reported for *A. mellifera *[22]. However, the tissue distribution, expression patterns and genomic structure of lepidopteran Tre-2 are still unknown. In the present study, Northern blotting and RT-PCR results suggest that *SeTre-2 *is expressed not only in midgut, but also in the fat body and Malpighian tubules (Figure 6). Moreover, *SeTre-2 *has different expression patterns in the midgut and fat body. *SeTre-2 *is expressed in the fat body, with higher expression levels in day-4 fifth instar larvae, and day-4 and day-7 pupae (Figure 7B). *SeTre-2 *transcripts were also detected in midgut throughout the feeding stage, which is consistent with results for *BmTre-2 *[21]. *SeTre-2 *expression levels before the wandering (pre-pupae) larval stage were higher than those in day-1–3 fifth instar larvae in both the midgut and fat body. A possible reason may be that more energy is needed for pupa development. According to preliminary results for RNAi experiments involving injection of dsRNA of an ecdysteroid receptor gene in *S. exigua*, changes in *SeTre-2 *transcripts in the midgut and fat body are modulated by morphogenetic hormones (data not shown).

Insect trehalases have several common characteristics, namely, a signal peptide leader, a coiled-coil domain, a highly conserved glycine-rich (GGGGEY) region, and two conserved signature motifs (Figures 1 and 2) [21]. In addition, Tre-2 also has some unique characteristics, such as a transmembrane helical region and two conserved motifs (DAKTFVDMK and LGRKM; Figure 2), but Tre-1 does not have a putative transmembrane region. Based on the genomic sequence of *S. exigua *obtained in this study, the exons and introns of *Tre-2 *are reported for the first time.

Trehalases are important enzymes in insects as they catalyze the hydrolysis of trehalose to glucose [13,14,17,18]. It has been reported that *B. mori *midgut contains two trehalases, BmTre-1 and BmTre-2 [21]. Tre-2 is involved in incorporating trehalose from the blood into muscular cells and then providing the energy required by visceral muscles to support peristaltic movement of the midgut for active feeding [13,21]. The chitin biosynthesis pathway starts with trehalose, which is mainly synthesized by trehalose-6-phosphate synthase in the fat body and released into the hemolymph in insects [20,34,35]. According to the *SeTre-2 *expression patterns observed in the midgut and fat body (Figure 7), Tre-2 may have different functions in these two tissues. This is the first report of trehalase transcript expression in fat body, but its function in this tissue is still unknown. We also cloned the trehalose-6-phosphate synthase gene (GenBank accession no. EF051258) from *S. exigua*, which is expressed mainly in fat body and not in midgut, and found that its expression levels showed the same trend as trehalose levels in hemolymph of *S. exigua *(unpublished data). This demonstrates that both Tre-2 and trehalose-6-phosphate synthase are synthesized in the fat body. Thus, the *Tre-2 *gene may have a crucial function in regulating the balance of trehalose in hemolymph.

The relative importance of Tre-1 and Tre-2 in the chitin biosynthesis pathway in *S. exigua *is currently being investigated in our laboratory.

## Conclusion

We have demonstrated that two trehalase genes exist in *S. exigua*. *SeTre-2 *transcripts are expressed not only in the midgut, but also in fat body and Malpighian tubules. Furthermore, there are different *SeTre-2 *expression patterns between midgut and fat body. This suggests that *SeTre-2 *may have different functions in these different tissues.

## Methods

### Insect cultures

*S. exigua *larvae were reared at 26 ± 1°C under a L14:D10 photoperiod on an artificial diet [36]. The developmental stages were synchronized at each molt by collecting new larvae or pupae. The midgut and fat body from larvae to pupae and the brain, cuticle, tracheae and Malpighian tubules from larvae were dissected in ice-cold saline, and stored at -80°C for later use.

### RNA isolation, cDNA synthesis and PCR

Total RNA was isolated from fat body of *S. exigua *pupae using an acid guanidinium thiocyanate-phenol-chloroform method [37,38]. Fat body (100 mg) was homogenized in solution D (solution D: 4M guanidinium thiocyanate, 0.025M sodium citrate, 0.1M mercaptorthanol, 0.05% sarcosyl), placed on ice for 5 min and then sodium acetate and chloroform/isoamylalcohol (49:1) were added. The sample was centrifuged at 10,000× *g *at 4°C for 20 min and the supernatant was transferred into a new tube, and isopropanol was then added. After centrifugation, the RNA pellet was washed in 75% ethanol and then dissolved in ddH_2_O. A sample of 1 μg of total RNA was reverse-transcribed at 42°C for 1 h in a 10-μl reaction mixture containing reaction buffer, 10 mM DTT, 0.5 mM dNTP, 0.5 mg of oligo-dT18, and reverse transcriptase from avian myeloblastosis virus (AMV, Takara, Japan) [39].

Three degenerate primers, SeTre-F1 (5'-CTA YTG GGA CDS WTA YTG G-3'), SeTre-F2 (5'-GCY GAR AGC GGK TGG GAC TT-3') and SeTre-R (5'-ACG CCR TTC GWC CAY CCG-3'), were designed based on the conserved amino acid sequences of known trehalases. The first PCR amplification was performed with primers SeTre-F1 and SeTre-R under the following conditions: 3 cycles of 40 s at 94°C, 40 s at 45°C, and 90 s at 72°C, then 28 cycles of 40 s at 94°C, 40 s at 48°C, and 90 s at 72°C. A second PCR was carried out using nested reverse primers SeTre-F2 and SeTre-R under the same conditions as for the first PCR. After PCR products were electrophoresed, a weak DNA band corresponding to the expected size of approximately 700 bp was excised from the agarose gel and purified using a DNA gel extraction kit (Takara, Japan). These PCR products were cloned into the pMD18-T vector (Takara) and sequenced by the dideoxynucleotide method (Takara).

### Rapid amplification of cDNA ends (RACE)

For 5'- and 3'-RACE, cDNA was synthesized according to the manufacturer's protocol (SMART™ kit, Clontech). Specific primers SeTreR1 (5'-CGG AGA AGC TGG GCG TTC C-3') and SeTreR2 (5'-GGC ATT CAG GTC TAC TGG G-3') for 5'-RACE, and SeTreF1 (5'-GGA CTA TCC GAA TGC CTG GC-3') and SeTreF2 (5'-CCA CTA AAT GGG TCA GGT CG-3') for 3'-RACE were synthesized based on the cDNA sequence obtained from the PCR product. 5'-RACE was performed on 2.5 μl of 5'-ready-cDNA with Universal Primer Mix (UPM, Clontech) and SeTreR1, then nested PCR was carried out with Nested Universal Primer (NUP, Clontech) and SeTreR2. 3'-RACE was performed on 2.5 μl of 3'-ready-cDNA with UPM and SeTreF1, then with NUP and SeTreF2. PCR conditions were 10 min at 94°C, followed by 30 cycles of 30 s at 94°C, 30 s at 55°C, and 90 s at 72°C, then 10 min at 72°C. After PCR products were electrophoresed, DNA bands corresponding to approximately 1200 bp from the 5'-RACE and ~600 bp from the 3'-RACE were excised from the agarose gel and purified using a DNA gel extraction kit (Takara, Japan). These PCR products were cloned into the pMD18-T vector (Takara) and sequenced by the dideoxynucleotide method (Takara). The resulting overlapping sequences were assembled to obtain the full-length *SeTre-2 *cDNA sequence. To confirm the assembled cDNA sequence from overlapping PCR products, the entire coding regions of *SeTre-2 *were amplified by PCR with the forward and reverse primers SeTreF5 (5'-CAT TGT CGA TAG TTT ATT TGT CG-3') and SeTreR3 (5'-CAC TCA CGT TCC ACC GGT CGA G-3'). PCR was performed as follows: denaturation at 94°C for 30 s, annealing at 55°C for 30 s and elongation at 72°C for 3 min using Takara *Taq *polymerase for 30 cycles.

### cDNA and protein sequence analyses

The sequence of *SeTre-2 *cDNA was compared with sequences of other trehalases deposited in GenBank using the BLAST-N and BLAST-X tools available from the National Center for Biotechnology Information (NCBI) web site. A phylogenetic tree was constructed using MEGA 3.1 software based on the amino acid sequences of known trehalases. A bootstrap analysis was carried out, and the robustness of each cluster was verified in 1000 replications. The amino acid sequence of *SeTre-2 *was deduced from the corresponding cDNA sequence using the translation tool at the ExPASy Proteomics website (please see Availability & requirements). Other protein sequence analysis tools used in this study, including molecular weight, p*I*, and N-glycosylation sites were also obtained from the ExPASy Proteomics website. The transmembrane helices of Trehalase proteins were analyzed using TMHMM v.2.0 (please see Availability & requirements). Multiple sequence alignments of deduced amino acid sequences were made using Vector NTI 9.0 software.

### Genomic DNA sequencing and gene structure analysis

To obtain the *SeTre-2 *gene, genomic DNA was extracted from the fat body of fifth instar larvae using a Genomic DNA Purification Kit (Promega) according to the manufacturer's instructions. Overlapping PCR fragments were obtained using pairs of gene-specific primers designed from the corresponding cDNA sequence of *SeTre-2 *and genomic DNA as a template. Cloning and sequencing of these PCR products were carried out in the same manner as described above [36]. Exons and introns were identified by comparing and analyzing the cDNA and genomic DNA sequences of *SeTre-2*.

### Southern blot analysis

Genomic DNA was prepared from fresh *S. exigua *pupae and was purified after complete digestion with *Hin*dIII, *Sal*I or *Xho*I. The digested DNA (15 μg per lane) was separated on a 0.8% agarose gel in TAE buffer (40 mM Tris acetate, 2 mM EDTA) After electrophoresis, DNA was transferred to Hybond-N^+ ^nylon membranes (Amersham) in 20× SSC [Au: What is SSC?] for 17 h [40]. DNA was fixed to the membrane by baking at 120°C for 30 min. A cDNA fragment of 770 bp (using primers SeTreFP 5'-AGG ATC TGA GTT CGA GGA CTG G-3' and SeTreRP 5'-GGC ATT CAG GTC TAC TGG G-3') was labeled with [α-^32^P]dCTP using a random primer DNA labeling kit (Takara) as the hybridization probe. Membranes were prehybridized at 42°C for 4 h, followed by addition of the ^32^P-labeled *SeTre-2 *cDNA at 42°C for 18 h in 5× SSPE (1× SSPE: 180 mM NaCl, 10 mM sodium phosphate, pH 7.7, 1 mM EDTA) containing 50% formamide, 5× Denhardt's solution, 0.1% SDS and 100 mg/ml salmon sperm DNA. After hybridization, the membrane was washed with 0.2× SSPE at 45°C, and finally exposed to X-ray film at -70°C for 24 h.

### Northern blot and RT-PCR analysis

To detect *SeTre-2 *expression in fat body, midgut and other tissues, Northern blotting and RT-PCR were performed. Samples of 25 μg of total RNA isolated from various larval tissues using TRIzol reagent (Life-Tech, Rockville, MD) were separated on a formaldehyde agarose gel containing ethidium bromide, and subsequently blotted onto a Hybond-N^+ ^membrane (Amersham). Membranes were prehybridized and hybridized with the [α-^32^P]dCTP-labeled probe as described above [41]. RT-PCR was performed using SeTreFP and SeTreRP primers and total RNA from midgut, brain, Malpighian tubules, cuticle, fat body, tracheae and spermary as templates for 30 cycles of 40 s at 94°C, 40 s at 55°C, and 60 s at 72°C. A 5-μl sample of each PCR product was electrophoresed and detected by ethidium bromide staining. β-Actin was used as a loading control.

### Developmental *SeTre-2 *expression in fat body and midgut

The fat body of fifth instar larvae, pre-pupae and pupae, and the midgut of third, fourth and fifth instar larvae, pre-pupae and pupae were dissected. Total RNA was isolated from the fat body of 12 stages and the midgut of 11 stages. Then 1 μg of total RNA from each sample was reverse-transcribed at 42°C for 1 h in a 10-μl reaction mixture containing reaction buffer, 10 mM DTT, 0.5 mM dNTP, 0.5 mg of oligo-dT18, and reverse transcriptase from avian myeloblastosis virus (AMV, Takara, Japan). PCR reactions were performed using primers SeTreFP, SeTreRP and SeActinF/R for 22 cycles of 40 s at 94°C, 40 s at 55°C, 60 s at 72°C. A 5-μl sample of each PCR product was electrophoresed and transferred to a Hybond-N^+ ^membrane and then hybridized with [α-^32^P]dCTP-labeled probes as described above. The amount of *S. exigua *β-actin loaded per lane is indicated as a control.

## Availability & requirements

ExPASy Proteomics: 

TMHMM v.2.0: 

## Authors' contributions

BT carried out most of experiments and co-wrote the manuscript. XC participated in *SeTre-2 *gene cloning and acquisition of data. YL performed data analysis and interpretation. HT performed protein sequence and data analysis. JL participated in the study design. JH have made substantial contributions to conception and design. WX have been involved in drafting the manuscript or revising it critically for important intellectual content. WZ conceived the project, supervised the experiments and co-wrote the manuscript. All authors read and approved the final manuscript.
